# Away with Legacy Duty for Voluntary Hospitals

**Published:** 1913-01-25

**Authors:** 


### AWAY WITH LEGACY DUTY FOR VOLUNTARY HOSPITALS.

### The Attitude of the Treasury.

It becomes increasingly plain that the British Treasury, of which the Chancellor of the Exchequer is the head, cannot successfully provide for the health necessities of the people of these islands, except at ruinous cost, without the continuous co-operation of the voluntary hospitals. For some unexplained reason the Treasury have steadily neglected the interests of the voluntary hospitals in recent years. So recently as May 1912 a very important and carefully drawn petition was addressed to the Chancellor of the Exchequer by the British Hospitals Association, as the representative of upwards of four hundred hospitals, which to this day has remained, we believe, without even an acknowledgment.

In another column we publish a copy of the second memorial addressed to the Chancellor of the Exchequer by the British Hospitals Association requesting him favourably to entertain the claim of these institutions to be relieved from the payment of legacy duty upon bequests made to them by benevolent persons. The prayer of this petition should receive immediate and most sympathetic attention at the hands of the Treasury.

The National Insurance Act, the benefits of which commenced last week, cannot secure for the insured, as matters stand at present, the most costly of all benefits, namely, that of adequate treatment in serious cases of illness or accident, except through the co-operation and at the cost of the voluntary hospitals. Further, and even more important in the financial sense from the Treasury's point of view, is the fact that, unless the voluntary hospitals immediately admit for treatment all these serious cases, the resulting delay in treatment must tend so enormously to increase the sick-pay claims upon approved societies as to undermine their financial strength and ultimately place them in liquidation. This startling fact is supported by the highest actuarial authority and must not be lost sight of. Even the British Treasury, with all its official aloofness, cannot and dare not ignore it. We shall watch, therefore, with great interest the reception by the Chancellor of the Exchequer of the second memorial addressed to him on the 15th inst. by the Council of the British Hospitals Association. Will it once again be treated without the courtesy of an acknowledgment, or will the important issues it possesses for the people of these islands be recognised, and prompt action taken upon it?

We are amongst those who have worked longest and believed most fully in the necessity for a wisely conceived and sympathetically administered system of National Insurance. It is painful and regrettable beyond measure to the writer to know of assured certainty that most of the deserved unpopularity, and practically all the many miseries afflicting and to be inflicted upon insured persons in the immediate future, are due to avoidable causes which could and ought to have been prevented by foresight, and statesmanlike care in the preparation of the Act and in the preliminary work preceding its coming into force. Benevolent persons throughout the country are pained and perplexed in mind by the misery they see around them resulting from these causes, and by the harsh treatment imposed upon voluntary hospitals through the maintenance of the legacy duty of 10 per cent. under conditions which are resented, we venture to say, by an overwhelming majority of benevolent people throughout the British Islands. It is not statesmanship, and what is more extraordinary still it is not good politics even, continuously to maintain a multitude of small and readily avoidable irritations, which collectively must so exasperate all thinking persons as to make them rise as one man to destroy the authors of these cruelties. Suicide is not supposed to be a favourite pursuit of politicians, but during the past twelve months scientists, at any rate, of every shade of opinion have come to feel that, if actions and conduct may be taken as symptoms, an epidemic of suicide has broken out amongst the members of the present Government without demonstrable cause.

### An Example to Donors.

The effects of the present epidemic of Governmental maladministration are making themselves felt in various ways. Those benevolent donors, accepting the attitude in which the present Government, or many members of it, present themselves to the community as deliberately hostile to the wishes and welfare of the people at large, are concerting measures in defence. Colonel J. H. Wilkinson has, for instance, sent a letter to the Birmingham and Midland Eye Hospital enclosing a cheque for £500 with a letter in which he states that he has provided in his will a legacy to this institution, the payment of which he now desires to anticipate. He has therefore placed this £500 at the Committee's immediate disposal, to be applied as they may deem fit. The Committee were, of course, immensely pleased by this action on Colonel Wilkinson's part, and decided to invest the £500 at once. We mention this case because, failing the generous consideration of the voluntary hospitals' plea for the remission of the legacy duty, which must commend itself to the mind of every living statesman in existing conditions, Colonel J. H. Wilkinson points the way whereby every generous donor who contemplates a legacy may give the full benefits his money can purchase directly to the voluntary hospitals by adopting a similar course to that just explained. Colonel Wilkinson's gift is certainly of an exceptional nature to-day, but should such a course become necessary we have little doubt that his example will be widely followed should the Chancellor of the Exchequer again ignore the opportunity to do prompt justice to the voluntary hospitals in this matter, which the petition just forwarded to him opportunely presents.

